# Detection of Reactive Oxygen and Nitrogen Species by Electron Paramagnetic Resonance (EPR) Technique

**DOI:** 10.3390/molecules22010181

**Published:** 2017-01-21

**Authors:** Sibel Suzen, Hande Gurer-Orhan, Luciano Saso

**Affiliations:** 1 Department of Pharmaceutical Chemistry, Faculty of Pharmacy, Ankara University, Tandogan, Ankara 06100, Turkey; 2 Department of Pharmaceutical Toxicology, Faculty of Pharmacy, Ege University, 35100 Izmir, Turkey; hgurer@gmail.com; 3 Department of Physiology and Pharmacology “Vittorio Erspamer”, Sapienza University of Rome, P. le Aldo Moro 5, 00185 Rome, Italy; luciano.saso@uniroma1.it

**Keywords:** free radical, oxidative stress, EPR, ROS, RNS

## Abstract

During the last decade there has been growing interest in physical-chemical oxidation processes and the behavior of free radicals in living systems. Radicals are known as intermediate species in a variety of biochemical reactions. Numerous techniques, assays and biomarkers have been used to measure reactive oxygen and nitrogen species (ROS and RNS), and to examine oxidative stress. However, many of these assays are not entirely satisfactory or are used inappropriately. The purpose of this chapter is to review current EPR (Electron Paramagnetic Resonance) spectroscopy methods for measuring ROS, RNS, and their secondary products, and to discuss the strengths and limitations of specific methodological approaches.

## 1. Introduction 

Reactive oxygen species (ROS) and reactive nitrogen species (RNS) are produced in almost all eukaryotic cells. These molecules are responsible regulating several physiological processes such as proliferation, migration, differentiation, and metabolism [1]. Excess amounts of ROS and RNS may react with vital molecules including lipids, proteins, and nucleic acids, thereby altering structural and functional properties of target molecules and leading to extensive tissue dysfunction and injury [2].

EPR (Electron Paramagnetic Resonance) spectroscopy is a very useful method for the direct detection of free radicals at concentrations as low as 1 µM. For short-lived ROS, the spin-trapping technique involves the addition of radicals to nitrone spin traps to form a spin adduct which has a relatively longer half-life to allow its detection using an EPR spectrometer. EPR enables the direct detection of free radicals, but other assays can be informative if used with proper controls [1]. EPR spin trapping has become an essential tool for the detection of ROS in biological systems. In this review the main benefits are described by the addition of some experimental considerations and applications to biological systems to measure oxidative stress (OS).

## 2. Free Radicals and Oxidative Stress

A free radical contains an unpaired electron (s) in its outer orbit. They are extremely reactive and easily take part in chemical reactions with vital cell components in the body. These reactions occur through a chain of oxidative reactions to cause tissue injury. ROS are the main cause of OS and are responsible for causing damage to proteins, lipids and DNA [3,4,5]. 

Although an excess amount of ROS may cause serious damage to cells, these reactive species play some important roles in a number of physiological processes. In mammalians, O_2_^−^ is known as the most commonly produced ROS [2]. The production of ROS by mammalian mitochondria is important since during its progress oxygen accepts one electron at a time, which leads to the generation of superoxide (O_2_^−^). The reduction of oxygen to form water occurs during the electron transport system of cellular respiration to lead to the generation of hydrogen peroxide (H_2_O_2_), and hydroxyl radicals (^•^OH). Although hydrogen peroxide by itself is unreactive, it oxidizes Fe(II) to Fe(III) to generate hydroxyl radicals which is called the Fenton reaction [6]. The hydroxyl radicals generated by the Fenton reaction are extremely reactive and short-lived. 

Both ROS and RNS are highly reactive, and as a result, they have short half-lives in biological environments. Therefore, these species are difficult to measure directly, and even indirect estimates of their abundance and reactivity are challenging. ROS/RNS are known to play a dual role in biological systems, since they can be either harmful or beneficial to living systems. The harmful effects of ROS are eliminated by the antioxidant achievement of non-enzymatic antioxidants in addition to antioxidant enzymes. Although many approaches have been adopted to bypass these limitations, and a wide range of methods have been developed to quantify ROS, RNS, and their secondary products, there are many considerations in the choice of assay and its application to a particular system. 

OS occurs by an imbalance between the production of ROS and a biological system’s ability to detoxify the reactive intermediates. OS may cause vital damage in cells which is related with the development of cancer, neurodegenerative diseases such as Alzheimer’s and Parkinson’s diseases, atherosclerosis, diabetes mellitus and so on [7,8]. OS also has been increasingly recognized as a contributing factor in various forms of pathophysiology related with aging. 

ROS have a significant role in a variety of cellular processes [9]. They are continuously produced during photosynthesis and respiration, whereas redox homeostasis in the cell is controlled by defensive mechanisms. Measuring OS in the cell requires delicate assays for ROS detection. Direct detection of ROS and RNS is quite difficult or sometimes impossible in solution at room temperature due to their very short half-life. Additionally, ROS are usually generated in subcellular compartments, which require detection procedures directed for specific localization. EPR allows researchers to detect ROS directly and it can also be used to monitor changes in the chemical forms of the oxidizable transition metal ions implicated in ROS generation [10]. EPR spectroscopy stands out from other methods because of its unique ability to detect either short- or long-lived radicals with high specificity and sensitivity. Correspondingly the technique of in vivo EPR spectroscopy can provide useful and even unique information pertinent to the study of oxygen/nitrogen radicals and related processes. Due to the low sensitivity of EPR, it is particularly challenging to measure ROS directly in vivo. The spin-trapping technique is used to overcome this problem. Using the spin-trapping method, ROS are allowed to react with specially selected trap molecules to produce less reactive and more stable species that can be readily detected by EPR [11]. 

## 3. Electron Paramagnetic Resonance (EPR)

EPR spectrometers are available in different sizes and models, dependent on the frequency and field in which they function. A microwave radiation source is needed for any EPR spectrometer. Microwave frequencies are produced at particular small frequency gaps called bands. The optimum sensitivity of EPR is the 8–12 GHz range which is called the X-band, a mid-range frequency. Numerous detection techniques exist for EPR spectroscopy. The most preferred EPR spectrometers are reflection spectrometers which measure the amount of radiation that is reflected back out of the resonator. A resonant cavity is the most common form of resonator used in EPR spectroscopy [12,13]. It is designed with dimensions corresponding to the specific wavelength of microwave radiation that is to be used. EPR experimentation needs an electromagnet that is used to organize the strength of the applied magnetic field. Furthermore, there are optional components which are commonly used in EPR experiments such as cooling devices to cool samples down to ultra-cold temperatures and radio frequency amplifiers to excite transitions of the nuclei. 

The continuous wave EPR (CW) method allows a sample to continuously illuminate with microwave radiation at a fixed frequency and detects changes in the microwave absorption. CW EPR is the most basic experiment and is widely used [14,15]. 

Pulse EPR varies from CW in that microwaves are applied to the sample as a series of nanosecond-long pulses. The magnetic field strength is kept constant, and the sample is pulsed with microwave energy. This technique involves the alignment of the net magnetization vector of the electron spins in a constant magnetic field [16].

Electron nuclear double resonance spectroscopy (ENDOR) is a technique that includes pulses in the radiofrequency region to the pulse patterns utilized to produce spin echoes. ENDOR spectroscopy employs both radiofrequency radiation, as in nuclear magnetic resonance (NMR) spectroscopy and microwave radiation, and as in electron spin resonance (ESR) or EPR, to study interactions between unpaired (paramagnetic) electrons and nuclei with magnetic moments [17].

## 4. Electron Paramagnetic Resonance (EPR) Spectroscopy Technique

EPR spectroscopy is a method for studying materials with unpaired electrons. The basic concepts of EPR are analogous to those of nuclear magnetic resonance (NMR), but it is electron spins that are excited instead of the spins of atomic nuclei. Instead of measuring the nuclear transitions in a sample, EPR detects the transitions of unpaired electrons in an applied magnetic field. 

EPR spectroscopy applications cover a wide range of areas from structural biology to quantum physics. EPR spin trapping is a technique that was first discovered in 1945 [18,19] and developed in the late 1960s. In this technique, the spin-trapping reaction occurs with the addition of the free radical to the double bond of a diamagnetic “spin trap”. After the formation of a more stable free radical, this can be examined with EPR. This technique detects species that have unpaired electrons such as free radicals and many transition metal ions. It is known that free radicals have a very short life but still play crucial roles in many processes in biological systems such as oxidation, catalysis, and polymerization reactions. 

EPR spectroscopy has been used to investigate nitric oxide chemistry and biology. Spin trapping is a well-known analytical technique widely used in chemistry and biology for the detection and identification of short-lived free radicals through the use of EPR. The most frequently used spin traps are alpha-phenyl *N*-tertiary-butyl nitrone (PBN) and 5,5-dimethyl-pyrroline *N*-oxide (DMPO). Secondly C-nitroso spin traps such as 3,5-Dibromo-4-nitrosobenzenesulfonic acid (DBNBS) can be preferred. 

EPR spectroscopy applications cover a wide range of areas from structural biology to quantum physics. The EPR characteristics can be obtained from EPR spectra of spin adducts such as the g-value, the hyperfine coupling constant (hfcc) and the spin concentration. The concentration of a free radical can be obtained as the double integrals of the EPR spectrum of a spin adduct which eventually allows us to investigate ROS and RNS qualitatively and quantitatively [18].

Khan and Swartz [20] described that in vivo EPR spectroscopy can provide valuable information relevant to the study of oxygen/nitrogen radicals. They pointed that there are some parameters that can be measured, including oxygen-, carbon- or sulfur-centered radicals, such as nitric oxide by spin trapping, oxygen by oxygen-sensitive paramagnetic materials and thiol groups using special nitroxides. The spin-trapping method by in vivo EPR is an effective technique for providing non-invasive measurements in animals. EPR imaging in the organs of live animals can also be observed after application of nitroxyl radicals as an imaging agent using EPR-computed tomography. In vivo EPR imaging has been established as an effective method for determining the free radicals in living organs and tissues [21]. EPR is a powerful technique when combined with an appropriate spin trap to measure O_2_^−^, ^•^OH, and NO in biological samples [1,22]. 

Rapid-scan EPR is based on continuous direct detection of the spin response as the magnetic field is scanned upfield and downfield through the resonance thousands of times per second. The method provides an improved signal-to-noise ratio for a wide range of samples, including immobilized radicals [23]. In contrast, conventional continuous wave (CW) EPR uses phase-sensitive detection at the modulation frequency. Recent developments of rapid-scan EPR provide an alternative way to observe EPR spectra. In rapid-scan EPR the signal is detected directly and the magnetic field is scanned through resonance in a time that is short relative to the electron spin relaxation times [24,25,26]. Rapid-scan EPR can also be used to extract relaxation information from a sample. 

## 5. Detection Examples of Reactive Oxygen and Nitrogen Species by EPR 

EPR spectroscopy can provide exclusive data on the identity, quantity, dynamics and environment of radical species. ROS and RNS play essential roles in regulating physiological and pathophysiological pathways in cells [27]. The direct or indirect observations of O_2_^•−^/HO_2_^•^ formation have been realized by spin trapping using biological systems such as mitochondria [28], nitric oxide synthase [29], endothelial cells [30] and human neutrophils [31]. The most popular cyclic nitrones can be stated as (DMPO) and 5-diethoxyphosphoryl-5-methyl pyrroline *N*-oxide (DEPMPO) [32] for similar studies.

The most commonly used spin traps for ROS detection are the pyrroline-based cyclic nitrones, such as DMPO and DEPMPO, which react with the OH and O_2_^−^ radicals to form –OH and –OOH adducts, respectively. It was found that cyclic hydroxylamines can react with O_2_^−^ to form more stable adducts compared to those with nitrone spin traps [33,34,35]. 

Recent studies have revealed that OS has important roles in various neuronal conditions such as stroke and traumatic brain injury. Dohi et al. [36] developed a technique for the detection of free radicals and OS using the ex vivo electron spin resonance (EPR) spin-trapping method in patients with neuroemergencies. The alkoxyl radical level was measured by ex vivo EPR spectrometry using DMPO as a spin trap in blood samples. This method was found useful and may prove valuable for clarifying the pathophysiology of neuroemergency diseases.

ROS, such as hydroxyl and superoxide radicals, have too-short lifetimes at ambient temperatures which are difficult to detect easily by EPR. When rapid-scan EPR is used, the magnetic field is scanned through resonance in a time that is short relative to the electron spin relaxation times, and data are processed to obtain the absorption spectrum. To validate the application of rapid-scan EPR to spin trapping, superoxide was generated by the reaction of xanthine oxidase and hypoxanthine and trapped with 5-*tert*-butoxycarbonyl-5-methyl-1-pyrroline-*N*-oxide (BMPO) by Mitchell et al. [37]. They observed that rapid-scan EPR can detect lower concentrations of BMPO-OOH radicals. Rapid-scan EPR will increase the chances to apply spin-trapping and stable nitroxides to explore important biological experiments in vivo and in vitro such as the detection of superoxide produced by Enterococcus faecalis at rates that are too low for detection by other methods.

Studies showed that the direct detection of some free radicals such as superoxide and hydroxyl radicals is quite problematic in solution at room temperature. The most popular spin trap is DMPO which has substantial benefits such as being most the redox-inactive. Commonly used nitrone spin traps, except DMPO, such as α-phenyl-*N*-tert-butylnitrone (PBN) and α-(4-pyridyl-1-oxide)-*N*-tert-butylnitrone (POBN), have EPR spectra of their radical adducts which show relatively little dependence on the structure of the trapped radicals. BMPO-derived adducts display a much higher signal-to-noise ratio in their EPR spectra, and this could be suitable for the detection of sulfite, hydroxyl and methyl radicals [38,39]. 

Recent developments have allowed short-lived ROS to be directly detected in solution using several methods. To study alternative spin configurations for important reactive intermediates such as oxenium ions, which are not very well known regarding their reactivity, lifetimes, and electronic configurations, a photo-precursor was synthesized to the m-dimethylamino phenyloxenium ion. This method achieved direct spectroscopic detection and EPR investigation of a ground-state triplet phenyl oxenium ion possible [40].

NO can be detected directly by EPR since it is a diatomic free radical. It is not possible to directly observe NO by EPR in any biological media. Several approaches have been established to trap and detect nitric oxide by EPR. One of them is diamagnetic spin trapping by using DMPO which can be used to trap superoxide, hydroxyl, and glutathionyl radicals. NO will not directly react with nitrone-based spin-traps to form spin adducts [41]. NO is considered a relatively stable radical that can conjugate with spin traps. Various studies define the use of EPR spin-trapping methods to detect NO in several in vitro and in vivo systems including in tumors [42,43]. Different iron compounds have been used to trap NO such as diethyldithiocarbamate (DETC) and *N*-methylglucamine dithiocarbamate (MGD). MGD was found specifically useful for the extracellular detection of NO while DETC is helpful for NO in the cellular lipid membrane [29,35].

Nitroxyl radicals display a one-electron reduction in reactions with oxidoreductases in mitochondria and microsomes, antioxidants and other free radical species in biological systems. After losing their paramagnetism with the one-electron reduction they primarily convert to the corresponding diamagnetic hydroxylamines [44]. A common OS producing such ROS is ionizing radiation. The β-ray irradiation can significantly decrease the EPR signal of a nitroxyl radical in a solution containing glutathione (GSH). In a study, the stability and reactivity of nitroxyl radicals in the reaction mixture containing an H-donor, such as GSH, NADH, or NADPH, were tested. It was found that the existence of an H-donor, such as GSH or NAD(P)H, was crucial to detect the reduction of nitroxyl radicals by low-dose irradiation. It was thought that another irreversible reaction of GSH and nitroxyl radicals was possible. The combination of 4-hydroxy-2,2,6,6-tetramethyl-1-piperidinyloxyl (4-hydroxy-TEMPO) and GSH was preferable for the quantitative detection of the free radical reaction caused by radiation [44]. 

Iron (II)-*N*-methyl-d-glucamine dithiocarbamate, Fe(MGD)_2_, is frequently used to trap NO due to making a large amount of stable adduct formation. In a study EPR spectroscopy was used to detect nitric oxide from bovine aortic endothelial cells and superoxide radical anions from human neutrophils using Fe(MGD)_2_ and 5,5-dimethyl-1-pyroroline-*N*-oxide, DMPO, respectively [45]. As a result, when the spin trapping of NO radicals was achieved using Fe^2+^-MGD, no EPR signal from Fe^2+^-MGD was found. When the DMPO spin trap was used for O_2_•-detection, DMPO did not show a signal, proving that the spin trap is free from paramagnetic impurities. The spectra of DMPO and PMNs (polymorphonuclear neutrophils), likewise, showed no detectable signal, suggesting that the DMPO does not cause activation of the enzyme NADPH oxidase. 

New cyclic nitrones with a triphenylphosphonium or permethylated β-cyclodextrin moiety have been synthesized and their spin adducts were demonstrated to increase stability in buffer which was studied in the presence of liver subcellular fractions. The stability and kinetics of the superoxide adducts of four cyclic nitrones were detected by the EPR spectrometer. The results proved that the new spin-trapping agents CD-DEPMPO and CD-DIPPMPO are suitable for the specific detection of superoxide, especially in the presence of liver microsomes [46,47].

Nitronyl nitroxides (NNs) are the paramagnetic probes that have the potential to scavenge ROS and RNS species and are used as probes for the detection of NO and HNO by EPR spectroscopy. Investigation of the redox properties of the two most frequently used NNs was stated in a study. The results showed that the rate constants of the reaction of the NNs with HNO were found to be very close to the rate constants of scavenging superoxide and NO. The scavenging rates of the NNs towards superoxide, NO, and HNO, and their low reduction potential being thermodynamically close to the bottom of the pecking order of oxidizing radicals were thought to be significant factors contributing to their antioxidant activity [48].

Nitrone spin traps have been used as probes for the determination of radical species in chemical and biological systems using EPR spectroscopy, and they have found pharmacological activity against OS-mediated diseases. Calix(4)pyrroles have shown to exhibit high affinity to (O_2_^•−^) anions; the cyclic nitrone conjugate of calix(4)pyrroles (CalixMPO) was designed by Kim et al. [32]. Computational studies showed that a pendant-type linkage between the calix(4)pyrrole and the nitrone was the most efficient design for the spin trapping of O_2_. The EPR method gave larger trapping rate constant for CalixMPO and a longer half-life for its O_2_^•−^ adduct compared to the commonly used nitrones. With these results the application of an anion receptor for the detection of one of the most important radical intermediates in biological and chemical systems was established. 

There are complementary methods for monitoring drug candidates. For example, the distribution and the pharmacokinetics of the new potential drug NOM (1-nitrosomelatonin) were evaluated. First [*O*-*methyl*-^3^H]NOM was administered to and followed in mice, and then monitoring of NOM, using EPR, was done in vitro and ex vivo with the (MGD)_2_–Fe^2+^ (iron–*N*-methyl-d-glucamine dithiocarbamate) complex as a spin trap. According to the characterization of the EPR signal in vitro with NOM or GSNO (S-nitrosoglutathione) using the (MGD)_2_–Fe^2+^ complex, the detection of these nitroso compounds was realized ex vivo in mouse tissue extracts. Results showed that NOM was able to cross the blood-brain barrier, while GSNO was not [49]. Studies showed that nitrosation may occur on thiol and indole derivatives giving nitroso compounds, which are in turn NO^•^ donors. Regarding thiols, GSNO and S-nitroso proteins have been identified in several biological samples [50,51]. 

NO can coordinate with both ferric and ferrous iron to form complexes. These complexes display EPR signals and so iron has been utilized in several ways as a spin trap for NO [52,53]. The ferrous iron complex is insoluble in aqueous media but water-soluble complexes have been applied for the biological detection of NO in vitro and in vivo [54]. One of the main benefits of using iron as a spin trap is that it will mostly react with NO very quickly [55].

An EPR technique using the spin probe cyclic hydroxylamine 1-hydroxy-3-methoxycarbonyl-2,2,5,5-tetramethylpyrrolidine (CMH) was introduced for the sensitive quantification of ROS, including the superoxide radical in frozen biological samples such as cell suspensions, blood or biopsies [56]. The absolute spin concentration in wet biological samples such as biopsies, water solutions and cell cultures could be quantified with higher precision and accuracy. This technique allowed researchers to collect and store the biological samples for future incubation with a spin probe, and also to further store them up to at least six months before EPR analysis, without loss of the signal intensity. 

In EPR experimentation, a common technique is to use a cell suspension to measure ROS in the cell. An in situ EPR detection methodology was developed to detect ROS produced in adherent cells. A quartz glass plate coated with poly-l-lysine (PLL) was used to improve the adhesion efficiency of cultured cells. This procedure was used for mouse fibroblasts and human malignant epithelial cells (HeLa) and significantly increased the EPR signal intensities of spin adducts related to superoxide radicals 1 h after the addition of a redox-active compound and a spin trap to the culture medium. These findings were demonstrated to be useful for measuring ROS generated under cell culture conditions [41]. Also, an EPR microinvasive method developed by Mrakic-Sposta et al. [57] provides direct evidence of the “instantaneous” presence of ROS, returning to absolute concentration levels that correlate with “a posteriori” assays of ROS-induced damage by means of biomarkers. The comparison of the results with antioxidant capacity and oxidative damage biomarker concentrations showed that all changes indicating increased OS are directly related to a ROS production increase. The method is noninvasive, sensitive, and quantitative, so it can be identified as an essential tool in the study of oxidants and OS in free radical biology and medicine.

## 6. Concluding Remarks

ROS are by-products of the aerobic metabolism of various pathological conditions. The EPR spectroscopy technique is able to deliver valuable information on the presence of ROS and RNS in biological systems. It is possible to derive the ultimate information on the identity of the species as well as information on their quantity, structure and possible interactions. 

There are some limitations for EPR spin trapping during ROS determination in vivo/in vitro, such as detection limit of 1 nM for O^2−^. The technique requires spin traps that reduce specificity and stability for the measurement [34]. EPR measurements usually require low temperatures and long recording times for ROS detection in physiological conditions [35,58]. The short lifetime of free radicals and the low rates of formation expected in in vivo detection by EPR are challenges. The new rapid-scan EPR method offers improved sensitivity for these types of samples. Making interpretations in complex and non-homogenous solutions is a key advantage of EPR compare to other spectroscopic techniques [59]. EPR techniques are capable of directly measuring ROS in vivo. Recent developments in analytical techniques such as EPR offer more accurate and quantitative approaches to measure ROS in cells. 
